# Association between Ankylosing Spondylitis and the miR-146a and miR-499 Polymorphisms

**DOI:** 10.1371/journal.pone.0122055

**Published:** 2015-04-02

**Authors:** Hui Ying Xu, Zhang Yang Wang, Jing Feng Chen, Tian Yang Wang, Ling Ling Wang, Li Li Tang, Xian-yang Lin, Chun-wu Zhang, Bi-cheng Chen

**Affiliations:** 1 Key Laboratory of Surgery, The First Affiliated Hospital of Wenzhou Medical University, Wenzhou, 325000, China; 2 Department of Surgery, The First Affiliated Hospital of Wenzhou Medical University, Wenzhou, 325000, China; 3 Injury Orthopaedics of Traditional Chinese medicine, The First Affiliated Hospital of Wenzhou Medical University, Wenzhou, 325000, China

## Abstract

miRNAs are small, non-coding RNAs that regulate the expression of multiple target genes at the post-transcriptional level. Single-nucleotide polymorphisms (SNPs) in miRNA sequences may alter miRNA expression and have been implicated in the pathogenesis of multiple forms of arthritis, including rheumatoid arthritis (RA) and osteoarthritis. The present study explored the association between ankylosing spondylitis (AS) and two single nucleotide polymorphisms (SNPs), miR-146a rs2910164G>C and miR-499 rs3746444T>C, in a Han Chinese population. A case–control study consisting of 102 subjects with AS and 105 healthy controls was designed. The two miRNA SNPs were identified by direct sequencing. Subsequently, their gene and genotype frequencies were compared with healthy controls. A significant difference was observed in the miR-146a rs2910164G>C SNP. The frequency of the G allele was markedly higher in the AS patients than in the healthy controls (*P* = 0.005, *P_c_* = 0.01, *OR* = 1.787), and the frequency of the GG genotype was higher in AS patients than in controls (*P* = 0.014, *P_c_* = 0.042, *OR* = 2.516). However, no significant association was found between the miR-499 rs3746444T>C variant and susceptibility to AS. This is the first study to address the association between the miR-146a rs2910164G>C and miR-499 rs3746444T>C polymorphisms and AS, and it suggests a potential pathogenic factor for AS. Further studies are needed to validate our findings in a larger series, as well as in other ethnic backgrounds.

## Introduction

Ankylosing spondylitis (AS) is a common inflammatory rheumatic disease that affects the axial skeleton, causing characteristic inflammatory back pain. It can lead to structural and functional impairments and a decrease in quality of life. Although the pathogenesis of AS remains ambiguous, it is likely that AS is a multifactorial disease, influenced by genetic and environmental factors. It is conceivable that the HLA-B27 gene is the most important risk factor for AS. However, many genes in and outside the MHC, as yet largely undefined, have also been demonstrated to be associated with the development of AS.

MicroRNAs (miRNAs, miRs) are a group of endogenous, 20–25 nucleotides long non-coding RNAs. Most miRNAs are transcribed by RNA polymerase II, and their upstream regulatory regions contain canonical core promoters and enhancers, regulated by transcription factors [1]. They function as posttranscriptional regulators of gene expression by specifically interacting with certain mRNAs and inducing their degradation or repressing their translation [2]. According to the miRBase database and other bioinformatic data, a mature miRNA can bind to many mRNA targets, and at least one-third of human protein-encoding genes appear to be regulated by miRNAs [3]. Thus, miRNAs have been implicated in a wide range of biological processes, including cell development, differentiation, proliferation and apoptosis [4]. Over the past several years, it has become increasingly clear that miRNAs are not only important for normal organismal development and physiology but also in the pathologies of autoimmune diseases, cancer, heart disease, and inflammation [5–7]. Regarding autoimmune diseases, it has been reported that many have similar underlying etiology and have shared susceptible genes [8]. Among all the miRNAs discovered, miR-146a and miR-499 have been received much attention in this field. miR-146a is among the most studied miRNAs and is encoded by chromosome 5q33. Mature miR-146a can bind to the 3’-untranslated regions of many target mRNAs, including interleukin-1 receptor-associated kinase 1 (IRAK-1), IRAK-2, tumor necrosis factor receptor-associated factor 6 (TRAF-6) and other transcripts associated with inflammatory signaling [9,10]. It has been proposed that miR-146a participates in Toll-like receptor and cytokine signaling [11], thus regulating the immune response. Accumulating evidence also suggests that miR-146a can be induced by NF-κB [12]. The miR-499 gene was mapped to 20q11.22 [13]. The targets of miR-499 include IL-17RB, IL-23a, IL-2RB, IL-6, IL-2, BTLA, IL-18R, IL-21, peptidyl arginine deiminase Type 4 (PADI4) and regulatory factor X 4 (RFX4, influences HLA class II expression) [14].

Single nucleotide polymorphisms (SNPs) are known to be the most common type of genetic variation in the human genome. SNPs located in miRNA regions can alter miRNA expression and/or maturation to affect function in three ways: through the transcription of the primary transcript, through pri-miRNA and pre-miRNA processing, and by affecting miRNA–mRNA interactions [15]. Recently, much effort has been made towards studying the role of SNPs in miR-146a and miR-499 and how these miRNAs may influence the normal activities of cells and the pathogenesis of many diseases. The common miR-146a polymorphism rs2910164 involves a G>C nucleotide replacement. It can lead to the change from a G:U pair to a C:U mismatch in the stem structure of the miR-146a precursor [8]. The miR-499 rs3746444T>C polymorphism has been found within the stem region of the miR-499 gene and results in an A:U to G:U mismatch in the stem structure of the miR-499 precursor [3]. Recent molecular epidemiologic research provided compelling evidence that the presence of these gene variations would be associated with to various diseases, such as inflammation, susceptibility to cancers, and the occurrence of type 2 diabetes [16,17].

It has been reported that miRNA expression could be altered in synovia, peripheral blood mononuclear cells (PBMCs) or T cells from patients with different forms of arthritis, including RA, OA and AS [18–21]. As mentioned above, SNPs located in a miRNA will give rise to its aberrant expression. It is plausible that there is a relationship between arthritis and certain SNPs. However, previous researchers have focused on the link between SNPs in miRNAs and RA or OA. None has ever studied the possible association between AS and miR-146a rs2910164G>C or miR-499 rs3746444T>C.

In our research, we hypothesized that miR-146a rs2910164G>C and miR-499 rs3746444T>C polymorphisms may be involved in the occurrence and development of AS. To test this hypothesis, the two SNPs were genotyped in patients with AS and healthy controls. Then, using statistical analysis, we evaluated the roles of these miRNAs in disease predisposition.

## Materials and Methods

### Study subjects

In total, 102 AS patients were enrolled in the study from the First Affiliated Hospital of Wenzhou Medical University between March 2013 and April 2014, and they were diagnosed strictly according to the modified New York criteria. The 83 male and 29 female AS patients were all *HLA-B27*-positive and ranged in age from 16 to 79 years, with an average age of 34.88 years. The 105 healthy controls, including 74 males and 31 females, were selected from the health examination center of our hospital. Their ages ranged from 17 to 55 years, with an average age of 32.14 years. This study was approved by the ethics committee of The First Affiliated Hospital of Wenzhou Medical University, approval number CR2014-070. Written informed consent was obtained from all participants. In a case of possible compromised capacity to consent, written informed consent was also obtained from a close relative of the participant. If the participant was a minor, both the participant and the next of kin provided written informed consent. This study was conducted according to the principles expressed in the Declaration of Helsinki.

### DNA extraction

Genomic DNA was extracted from EDTA anticoagulant blood using the DNAfast2000 DNA Extraction Kit (Fastagen, Shanghai, China) according to the manufacturer’s instructions. The concentration and purity of DNA were measured using ultraviolet (UV) spectrophotometry (Eppendorf). The extracted DNA was detected immediately or stored at -20°C for less than 6 months.

### Genotyping

A total of 207 individuals were genotyped for the two SNPs (rs2910164 G>C and rs3746444 T>C) by directly sequencing. The primers used for rs2910164 were 5-CACCCACATCAGCC TTCCAG-3 and 5-CTCCAGGTCCTCAAGCCCAC-3, and the primers used for rs3746444 were 5-ACTTCCCTGCCAAATCCC-3 and 5-GTTCCAGACGGTGTCCCA-3. The polymerase chain reaction (PCR) conditions for rs2910164 were 95°C for 3 min followed by 40 cycles of 95°C for 5 s and 60°C for 30 s, and a final 2 min extension at 70°C. The PCR conditions for rs3746444 were 95°C for 3 min, 40 cycles of 95°C for 10 s, 60°C for 15 s, and 72°C for 15 s, with a final extension at 72°C for 10 min. The PCR products were sent to GENEMIZ for sequencing to detect the SNPs.

### Statistical analysis

Statistical analyses were conducted using SPSS 15.0. The genotype and allele frequencies of the AS patients and healthy controls were compared using the Chi-squared test or Fisher’s exact test. The genotype distribution was evaluated using the asymptotic Pearson’s v2 test to determine whether it met the conditions of Hardy–Weinberg equilibrium (HWE). The power was calculated using PASS11.0 (a power value >80% was considered as high power to detect the significant difference). The Bonferroni method was used to correct the data. *P*<0.05 was considered statistically significant, and all statistical tests were two-sided.

## Results

### Analysis of the allele frequency

In total, 102 AS patients and 105 healthy individuals were successfully genotyped for their rs2910164 G/C and rs3746444 T/C SNPs. As shown in Table 1, we found a significant association between the miR-146a rs2910164 G>C variant and AS patients. Among the AS patients, the G allele frequency was clearly higher than in the healthy controls (49.0% vs. 35.2%, *P* = 0.005, *Pc* = 0.01, *OR* = 1.767). However, the miR-499 rs3746444 T/C SNP showed no association with the susceptibility of AS at the allele level (75.0% vs. 85.2%, *P* = 0.369, power = 15%).

**Table 1:** Allelic frequencies of the has-miR-146a and has-miR-499 variants in AS patients and healthy controls.

Allele	AS patients (2n = 204)	Healthy controls (2n = 210)	*P* value	*P*_*c*_ value	OR (95%CI)
MiR-146a rs2910164					
G	100	74	0.005	0.01	1.767 (1.191–2.261)
C	104	136	0.005	0.01	0.566 (0.381–0839)
MiR-499 rs3746444					
T	180	179	0.369	0.728	1.299 (0.733–2.301)
C	24	31	0.369	0.728	0.770 (0.435–1.364)

The frequency of the miR-146a rs2910164 G allele was much higher in AS patients (*P* = 0.005, *P*_*c*_ *=* 0.01). However, there was no association between the miR-499 rs3746444 T/C variant and AS (P>0.05).

*P*_*c*_: Bonferroni-corrected *P* value.

### Analysis of genotype frequency distribution

The distributions of the genotype frequencies for the two SNPs complied with Hardy-Weinberg equilibrium in both the cases and controls (*P*>0.05, data not shown). There was no population stratification and no sampling bias. As shown in Table 2, we found a significant association between miRNA-146a rs2910164 and the risk of AS. The frequency of the GG genotype was much higher in AS patients than in healthy controls (GG vs. GC+CC, *P* = 0.014, *P*_*c*_ = 0.042, *OR* = 2.516). However, no difference was observed in the distribution of miR-499 rs3746444 T>C genotypes between patients with AS and controls (*P*_*c*_>0.05, power = 37%).

**Table 2:** Genotypic frequencies of the has-miR-146a and has-miR-499 variants in AS patients and healthy controls.

Genotype	AS patients (n = 102)	Healthy controls (n = 105)	*P* value	*P*_*c*_ value	OR (95%CI)
MiR-146a rs2910164					
GG	25	12	0.014	0.042	2.516 (1.187–5.336)
GC	50	49	0.753	/	1.099 (0.637–1.896)
CC	27	44	0.019	0.057	0.499 (0.278–0.897)
MiR-499 rs3746444					
TT	79	74	0.253	0.759	1.439 (0.770–2.690)
TC	22	31	0.190	0.570	0.656 (0.349–1.234)
CC	1	0	0.990	/	0.990 (0.971–1.010)

The frequency of miR-146a rs2910164 GG was much higher in AS patients than in healthy controls (*P* = 0.014, *P*_*c*_ = 0.042). No association existed between the genotype of miR-499 rs3746444 and AS.

*P*_*c*_: Bonferroni-corrected *P* value.

### Association between microRNA polymorphisms and AS activity parameters

**Fig 1:**
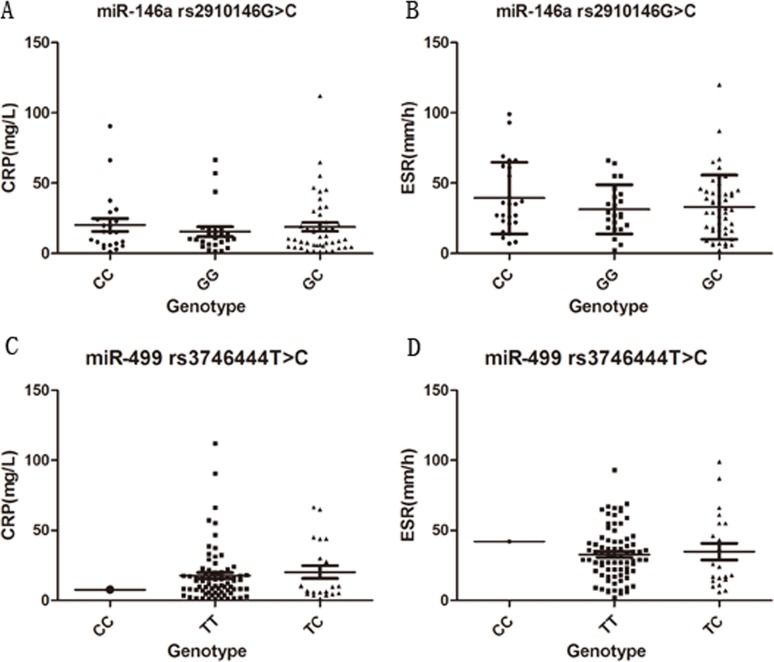
Association between variants of miR-146a (rs2910164) and miR-499 (rs3746444) and activity parameters. A. The levels of CRP among different miR-146a genotypes. No significant differences were found (CC vs. GG, *P* = 0.43; CC vs. GC, *P* = 0.79; GG vs. GC, *P* = 0.515). B. The levels of ESR among miR-146a genotypes. There were no significant differences among the three genotypes (CC vs. GG, *P* = 0.222; CC vs. GC, *P* = 0.249; GG vs. GC, *P* = 0.782). C. The levels of CRP among different miR-499 genotypes. No significant differences were found (TT vs. TC, *P* = 0.607). D. The levels of ESR among miR-499 genotypes. There were no significant differences (TT vs. TC, *P* = 0.759).

As shown in Fig. 1, there were no significant differences in the levels of CRP and ESR among different miR-146a genotype groups of AS patients, nor were there differences among miR-499 polymorphism groups. The two miRNA polymorphisms had no association with the disease activity of AS.

## Discussion

The study of miRNA biology has attracted increasing attention, resulting in rapid advances in this field. SNPs in the miR gene region may directly affect the expression of the mature miRNA, resulting in diverse functional consequences. To date, various epidemiological studies have indicated associations between SNPs in miRNAs and cancer susceptibility [3], autoimmune diseases [8,14], schizophrenia [22] and cardiovascular disease [19,23].

In the present work, we found that the miR-146a rs2910164 variant was associated with increased risk of AS at both the genotypic and allelic levels. The G allele and GG genotype were more prevalent in the patients than in the controls. Nonetheless, the miR-499 rs3746444 T>C variant was not associated with the risk of AS. To our knowledge, our study provides the first evidence that miR-146a rs2910164 G>C can serve a potential diagnostic biomarker for AS.

This common polymorphism, rs2910164 G/C in miR-146a, is located on the stem region, opposite from the mature miR-146a sequence [13]. It can cause a C:U mismatch in the miR-146a precursor [24]. For two reasons, many people hold the view that the G allele confers a higher expression level of the mature miR-146a: 1) The change from C to G may increase the stability of the miR-146a. One study showed that the optimal free energy of miR-146a differed between people with the G allele or the C allele [17]. The free energy of the complementary strand with the G allele was determined to be—26.8 kcal/mol, whereas that of the C allele was—24.0 kcal/mol. This indicates that this SNP may affect the stability of the miRNA, thereby influencing its expression level [6]. 2) The G allele displayed increased production of mature miR-146a compared with the C allele because the C allele was found to have lower transcriptional activity and attenuate the processing of pri-miR-146a [24,25]. By contrast, Shen et al [26] declared that the binding capacity was statistically significantly stronger in the variant C allele than in the common G allele using an in vitro assay, and that patients with breast or ovarian cancer and the variant C allele miR-146a may have high levels of mature miR-146. Therefore, it remains unclear which allele is associated with a significantly higher level of mature miR-146a. To clarify this discrepancy, further studies on the relationship between genetic variations and mature miRNA expression are warranted. The pathogenesis of AS has still not been fully identified. It has been suggested that aberrant expression levels of miR-146a may be linked to the development of AS and its clinical states [27], but the underlying mechanisms are not fully understood. We speculate that the inappropriate regulation of the inflammatory response may play a role in the development of this disease. Previous studies in AS patients have detected increased protein expression of TLR-4 in peripheral blood mononuclear cells, especially monocytes [27,28]. There has been an established theory that, like the recognition of TLR2 and TLR4, miR-146a expression may be induced by lipopolysaccharide and pro-inflammatory mediators through the NF-κB activation pathway [29]. TRAF6 and IRAK1 are two targets of miR-146a, which is involved in inflammation. miR-146a can impair NF-κB activity by down-regulating these two targets, finally inhibiting the expression of NF-κB target genes such as IL-6, IL-8, interleukin-1β (IL-1β), and TNF-α [16,29,30]. In this context, miR-146a would act as an effective regulator to prevent an overstimulated inflammatory state. Reduced expression of miR-146a could contribute to the prolonged production of inflammatory cytokines through the NF-κB pathway. Meanwhile, NF-κB DNA-binding activity could consistently increase in lymphocytes, even after several months of adequate therapy [31]. It is believed that in the development of AS, receptor activator of NF-κB ligand (RANKL) and a variety of pro-inflammatory cytokines, including IL-1β, IL-17, and TNF-α, contribute to the increased osteoclast activity and lead to bone destruction in inflamed joints [32].

Recently, several studies of AS patients have also suggested that T cells may play an essential role in the immunopathogenesis of AS, including T cell expansion, monocyte infiltration in synovia, and imbalances between CD4+T cells (Th1/Th2/Treg/Th17) [27,33,34]. According to previous studies, miR-146a may modulate many aspects of T cell-associated immunity. Thus, a causal link between AS and miR-146a may be easily established. For example, in cell fate determination and differentiation, studies have shown that miR-146 expression is elevated in Th1 cells but downregulated in Th2 cells, and it can promote Th1 differentiation [35,36]. In T cell activation, researchers have indicated that miR-146a–deficient T cells were hyperactive in chronic inflammatory response in vivo and promoted the development of T cell–associated autoimmunity [37].

Regarding miR-499, although there is no literature directly focusing on the role of miR-499 in the pathogenesis and development of AS, it can be speculated that a certain mutuality does exist. On the one hand, it has been demonstrated that IL-17RB, IL-23a, IL-2RB, IL-6, IL-2, and IL-18R are targets of miR-499 [14], and these cytokines have been shown to play multiple roles in AS [17,38]. On the other hand, studies have shown that HLA-DRB1 has significant associations with autoimmune diseases [39] and regulatory factor X 4 (RFX4). As one target of miR-499, RFX4 can influence the expression of HLA-DRB1. Therefore, miR-499 may regulate HLA-DRB1 via the RFX4 pathway and participate in the pathogenesis of autoimmune diseases[4]. AS is an incurable autoimmune disease. Therefore, miR-499 may trigger the inflammatory response and induce the pathogenesis and development of AS. Unfortunately, our study did not detect any association between miR-499 polymorphism and AS. However, it is still too early to assert that this miRNA has no association with AS. The negative results may have been due to the small sample size of our study (power <80% at both the allelic and genotypic levels) and the imbalanced distribution of TC and CC genotypes, so replication of the experiment in larger samples will be needed. Further, some unidentified mechanisms may confound the impact caused by miR-499 rs3746444 T>C.

In summary, this is the first report demonstrating the relationship between miR-146a rs2910164 G>C and AS. We demonstrate that G alleles in the locus are associated with an increased risk for AS. This SNP may serve as a promising candidate marker for identification of AS. Furthermore, we hypothesize that this SNP may also be useful for identifying healthy people who are more prone to develop inflammatory disease. Further larger investigations are needed to verify this conclusion. If this is the case, an assessment of the rs2910164 during examinations of high-risk patients could lead to the early detection of AS. Whether the miR-499 rs3746444 gene is a non-significant factor in AS requires further validation. Our research sheds new light on the significant relationship between natural genetic variations in miRNA genes and human diseases. Studies with large cohorts and diverse ethnicities are warranted to validate our finding. In this field, further investigation on the expression level of miRNAs and different factors in the signal pathway will gradually reveal the underlying pathogenic mechanism of AS.
